# Using the Lives Saved Tool (*LiST*) to Model mHealth Impact on Neonatal Survival in Resource-Limited Settings

**DOI:** 10.1371/journal.pone.0102224

**Published:** 2014-07-11

**Authors:** Youngji Jo, Alain B. Labrique, Amnesty E. Lefevre, Garrett Mehl, Teresa Pfaff, Neff Walker, Ingrid K. Friberg

**Affiliations:** 1 Department of International Health, Johns Hopkins Bloomberg School of Public Health, Baltimore, Maryland, United States of America; 2 Johns Hopkins University Global mHealth Initiative, Baltimore, Maryland, United States of America; 3 World Health Organization, Department of Reproductive Health and Research, Geneva, Switzerland; 4 Department of Community Public Health, Johns Hopkins School of Nursing, Baltimore, Maryland, United States of America; School of Population Health, The University of Queensland, Australia

## Abstract

While the importance of mHealth scale-up has been broadly emphasized in the mHealth community, it is necessary to guide scale up efforts and investment in ways to help achieve the mortality reduction targets set by global calls to action such as the Millennium Development Goals, not merely to expand programs. We used the Lives Saved Tool (*LiST*)–an evidence-based modeling software–to identify priority areas for maternal and neonatal health services, by formulating six individual and combined interventions scenarios for two countries, Bangladesh and Uganda. Our findings show that *skilled birth attendance and increased facility delivery* as targets for mHealth strategies are likely to provide the biggest mortality impact relative to other intervention scenarios. Although further validation of this model is desirable, tools such as *LiST* can help us leverage the benefit of mHealth by articulating the most appropriate delivery points in the continuum of care to save lives.

## Introduction

Globally, nearly 3.5 million babies die each year in their first month of life, accounting for about 40–50% of under-five-child deaths. [1], [2] These deaths are from largely preventable or treatable conditions such as birth asphyxia, prematurity and neonatal infections. [3] While Millennium Development Goals (MDGs) 4 has set country targets to reduce the under-five mortality rates (U5MR) by two thirds by 2015, governments are seeking to identify promising ways to improve the delivery of effective life-saving interventions, often within the context of limited financial and human resources.

mHealth, the facilitation of improved healthcare services, health outcomes and provision of information via mobile and wireless technologies [4], has created a unique opportunity to transform the way in which global health challenges can be tackled. At the end of 2013, there were more than 6.8 billion mobile subscriptions worldwide, with 89% of them in developing countries. [5] Most people living on no more than USD 1 per day have access to these ubiquitous mobile phones, which have leapfrogged the pace of conventional landline infrastructure development. Numerous organizations have recognized the potential of harnessing mobile platforms [6] and have begun to explore ways to employ mHealth innovations to improve the delivery of maternal and neonatal health (MNH) interventions and practices. Sparse resources, vast populations and numerous socio-cultural barriers have challenged continued progress towards the MDGs in many settings, specifically MDGs 4 and 5, which are lagging behind. [7] Among the persistent health system challenges to improving these key maternal and newborn indicators are the lack of timely and actionable disease surveillance, a shortage of professional health workers, delays throughout the health delivery system, poor supply chain management and use of counterfeit drugs. [8] Driving the many experiments with mHealth is a belief that such strategies can help to overcome the health system challenges through improved access, transparency, and accountability while reducing costs and time. [9] mHealth initiatives also show promise in reaching underserved populations, particularly those in the developing world, changing health behaviors and outcomes, and addressing a wide variety of healthcare challenges. [10] The mobile platform presents the unique capability to strengthen the role of community health workers (CHWs) to deliver higher quality healthcare services wherever people are–not just in healthcare facilities [11].

### mHealth for community health workers on maternal and newborn health services

Capitalizing on the ability of mobile technologies to facilitate communication and remove barriers to healthcare, mHealth can support critical interactions between CHWs and families. [12], [13] A review of recent mHealth approaches and examples of MNH interventions leveraging mobile technologies produced a number of illustrative examples. [14] [File S1] Projects have been selected based on demonstrated lessons, benefit or impact evidence in coverage improvements; these illustrative examples of strategies used across the MNH continuum have been summarized in Table 1.

**Table 1:** Examples of mHealth programs on MNH services through CHWs.

Project	Country	Organization	Interventions	mHealth strategies	mHealth benefit/impact evidence on service provision
Wired Mothers	Tanzania	DanishInternationalDevelopmentCooperation,University ofCopenhagen	(i) Family planning (ii) Behaviorchanges through Information,Education and Communication(IEC) (iii) Antenatalcare(ANC)/Expanded Program onImmunization (EPI)/Postnatalcare(PNC) (iv) Skilled birthattendance(SBA)/Facility delivery(FD)	(i) Datacollection and management(e.g. Risk assessmentand classification,Vital events tracking,adherence reminder)(ii) SMS texting forhealth promotion andscheduled visitsreminder(with mobile phone vouchercomponents)	“The mobile phone intervention was associated with an increase in antenatal care attendance. In the intervention group 44% of the women received four or more antenatal care visits versus 31% in the control group (odds ratio (OR), 2.39; 95% confidence interval (CI), 1.03–5.55). There was a trend towards improved timing and quality of antenatal care services across all secondary outcome measures although not statistically significant.” [22]
					“The mobile phone intervention was associated with an increase in skilled delivery attendance: 60% of the women in the intervention group versus 47% in the control group delivered with skilled attendance. The intervention produced a significant increase in skilled delivery attendance amongst urban women (OR, 5.73; 95% CI, 1.51–21.81), but did not reach rural women.” [34]
					“The perinatal mortality rate was lower in the intervention clusters, 19 per 1000 births, than in the control clusters, 36 per 1000 births. The intervention was associated with a significant reduction in perinatal mortality with an OR of 0.50 (95% CI 0.27–0.93). Other secondary outcomes showed an insignificant reduction in stillbirth (OR 0.65, 95% CI 0.34–1.24) and an insignificant reduction in death within the first 42 days of life (OR 0.79, 95% CI 0.36–1.74).” [40]
MaternalandNewbornHealth inEthiopiaPartnership(MaNHEP)	Ethiopia	UniversityResearch Co.,LLC, QualityImprovement Advisor forthe Maternaland NewbornHealth inEthiopiaPartnership	(i) Family planning(ii) Behavior changesthrough IEC(iii) ANC/EPI/PNC	(i) SMS texting forhealth promotion andscheduled visitsreminder (e.g.promotion ofcommunity maternaland newborn healthfamily meetings andlabor and birthnotification)	“Women who had additionally attended 2 or more CMNH meetings with family members and had access to a health extension worker’s mobile phone number were 4.9 times more likely to have received postnatal care (OR, 4.86; 95% CI, 2.67–8.86; *P* _.001).” [41] “Notification of health extension workers for labor and birth within 48 hours was closely linked with receipt of postnatal care. Women with any antenatal care were 1.7 times more likely to have had a postnatal care visit (OR, 1.67; 95%; 95% CI 1.10–2.54; *P* _.001).” [41]
E-IMCI	Tanzania	D-Tree	(i) ANC/EPI/PNC (ii) Behavior changes through IEC	(i) Point of care decision support through compliance to IMCI protocols	“For all ten critical IMCI items included in both systems, adherence to the protocol was greater for eIMCI than for pIMCI. The proportion assessed under pIMCI ranged from 61% to 98% compared to 92% to 100% under eIMCI (p<0.05 for each of the ten assessment items).” [25]
ProjectMwana	Zambia, Malawi	UNICEF	(i) HIV-antiretroviral therapy (ART) surveillance and treatment	(i) Datacollection and management(ii) SMS texting for healthpromotion and scheduledvisits reminder	“ SMS delivery of results can increase turnaround times by 50% on average, with a greater positive impact in rural facilities” [42]
BetterBorderHealthcareProgram	Thailand-Burma	MahidolUniversity,Thailand	(i) Family planning (ii) ANC/EPI/PNC	(i) Datacollection and management(ii) SMS texting for healthpromotion andscheduled visits reminder	“ANC/EPI coverage in the study area along the country border improved; numbers of ANC and EPI visits on-time as per schedule significantly increased; there was less delay of antenatal visits and immunizations” [43]
RapidSMS-MCH	Uganda	Ministry ofHealthUganda,UNICEF	(i) Family planning(ii) Behavior changesthrough IEC(iii) ANC/EPI/PNC(iv) SBA/FD	(i) Datacollection and management(ii) SMS texting for healthpromotion andscheduled visits reminder	Study reported “a 27% increase in facility based delivery from 72% twelve months before to 92% at the end of the twelve months pilot phase.” [44]
Rural Extended Services and Care for Ultimate Emergency Relief (RESCUER)	Uganda	Ministry of Health, UN Population Fund and the Uganda Population Secretariat	(i) Behavior changes through IEC(ii) ANC/EPI/PNC(iii) -SBA/FD	(i) Emergency medical referral (e.g. referral calling) with transportation services	“improved communication and transportation links between the Traditional Birth Attendants (TBAs) and the health posts resulted in increased and more timely referrals as well as the improved delivery of healthcare to a large number of pregnant women”… “The increased number of deliveries under trained personnel and increased referrals to health units led to a reduction of about 50 percent in the maternal mortality rate (MMR) in three years” [45]
M4RH	Kenya, Tanzania	USAID, FHI 360’s PROGRESS (Program Research for Strengthening Services)	(i) Family planning(ii) Behavior changesthrough IEC	(i) Datacollection and management(ii) SMS texting for health promotion and scheduled visits reminder	User interviews reported various positive responses including “the text messaging service was perceived as being private, convenient, and cost-effective.” [33]
PREVEN	Peru	Cell-Preven	(i) Sexual andreproductive healthsurveillance andservice delivery	(i) Datacollection and management(ii) SMS texting forhealth promotion andscheduled visits reminder	Lessons include “Two-way information systems are more than just collecting data. They provide feedback and support to health care workers in the field. Many times, only managers have information that allows them to monitor and evaluate data but these systems do not prove any aggregate value to health care workers in the field. A well-designed information system has to support and enhance the performance of all user levels in a secure environment.” [30] “Prahalad (2005) has reported that health workers in some developing countries spend as much as 40% of their time filling out forms, compiling and copying data from different pro-grams (e.g., tuberculosis, malaria, HIV/AIDS, etc.). By choosing the most appropriate information technology, we can avoid duplication and deploy different devices–i.e., cell phones, Internet–to report from each public health program.” [30]
Aceh Besar Midwives	Indonesia	UNICEF, UNFPA, andWorld Vision	(i) Behavior changesthrough IEC(ii) ANC/EPI/PNC(iii) SBA/FD	(i) Data collectionand management(ii) SMS texting for health promotion and scheduled visits reminder(iii) Emergency medical referral(e.g. referral calling)	“Findings from the project indicate that the mobile phone has proven to be an effective and efficient device for facilitating smoother communication, and allowing speedier emergency response. The system also aids in gathering and disseminating health-related information to midwives, who in turn convey this knowledge to the patient community.” [13]
MAMA	Bangladesh, India, and South Africa	mHealth Alliance	(i) Family planning(ii) Behavior changesthrough IEC	(i) Data collectionand management(ii) SMS texting for health promotionand scheduledvisits reminder	MAMA Bangladesh Aponjon project represented “a 37% increase over a 2011 national baseline of 26% attending four ANC visits. It is also important to note that 45% of the Aponjon subscribers went to a facility for delivery and 32% chose safe delivery at home” [32]
MOTECH	Ghana	Grameen Foundation	(i) Family planning(ii) Behavior changes through IEC(iii) ANC/EPI/PNC(iv) SBA/FD	(i) Data collectionand management(ii) SMS texting for health promotionand scheduled visits reminder(iii) Emergency medical referral(e.g. referral calling)	Comprehensive observational studies demonstrated lessons learned and key future implications. [28] Evaluation is on-going with Grameen Foundation, Healthcare Innovation Technology LAB (HITLAB), and Ghana’s School of Public Health. [29]

SOURCE: Compiled from references [13], [22], [25], [28]–[29], [30]–[34], [40]–[45].

Numerous mHealth strategies for CHWs leverage mobile phone connectivity and computing capacity to enable task shifting, bolster core skills, enhance outreach and facilitate referral, while improving data collection and transmission. [15] [16] Key examples of mHealth platforms, engaging CHWs delivering MNH interventions, involve data collection and management (e.g. risk assessment and classification, vital event tracking) [17] [18] [19], Short Message Service (SMS) texting for health promotion and scheduled visits reminder [20] [21] [22], emergency medical referral [23] and point of care support [24] [25], often through two simple functions, voice communication [26] and texting [27], and sometimes with customized applications, linked to more complex back-end, or server-side, messaging and information services. To help CHWs register and identify women and newborns in their area who need healthcare services, Mobile Technology for Community Health (MOTECH) [28] [29] and Cell-PREVEN [30] use electronic handheld devices for texting or data collection and management through mobile phones or personalized digital assistants (PDA), automating the process of tracking patients who have received care. During the maternal postpartum/neonatal postnatal period, Mobile Alliance for Maternal Action (MAMA) [27], [31] [32] and Mobile for Reproductive Health (m4RH) [33] use voice communication and SMS texting to remind scheduled antenatal care (ANC) or postnatal care (PNC) visits and to promote behavior change and communication messages (BCC) in the antenatal/early pregnancy period. At birth, Wired Mother uses SMS reminder and mobile phone voucher component to promote facility delivery. [34] In this way, these programs help improve health-seeking and preventative behaviors of pregnant women, new mothers and their families, such as skilled birth attendance, immediate exclusive breast feeding, wrapping of the newborn, clean postnatal practices, and danger sign recognition. To improve child health and reduce childhood mortality and morbidity, Interactive Research and Development (IRD) [35] and mTikka [36] use text messaging systems with cash incentives to facilitate routine immunization programs, sending reminders to registered parents when their child is due for immunization and/or to provide health promotion notifications for immunization campaign days. These mHealth applications, such as Electronic Integrated Management of Childhood Illness (eIMCI), have also been utilized to guide point of care support in which CHWs adhere step by step to Integrated Management of Childhood Illness (IMCI) protocols, a standardized strategy developed by the World Health Organization (WHO) and UNICEF to reduce mortality and morbidity among children younger than 5 years of age [37] [38].

While there has been a growing body of evidence that has documented the benefit of mHealth as exploratory or observational studies, few studies systematically demonstrated effectiveness of mHealth strategies to promote MNH service delivery. Free et al conducted a comprehensive systematic review and meta-analysis on the effectiveness of mobile health technologies to improve health care service delivery processes. The findings demonstrated that the pooled effect on appointment attendance using SMS reminders vs no reminder was increased, with a relative risk (RR) of 1.06 (95% confidence interval (CI), 1.05–1.07). [39] Wired Mothers, based on a clustered randomized controlled trial in Tanzania, recently demonstrated a positive effectiveness of mHealth in improving ANC attendance (odds ratio (OR), 2.39; 95% CI, 1.03–5.55) [22], skilled birth attendance (SBA) utilization (OR, 5.73; 95% CI, 1.51–21.81) [34], and perinatal mortality reduction (OR, 0.50; 95% CI, 0.27–0.93). [40] Maternal and Newborn Health in Ethiopia Partnership (MaNHEP) stated, “Women who had additionally attended 2 or more community MNH meetings with family members and had access to a health extension worker’s mobile phone number were 4.9 times more likely to have received postnatal care (OR, 4.86; 95% CI, 2.67–8.86).” and “Notification of health extension workers for labor and birth within 48 hours was closely linked with receipt of postnatal care. Women with any antenatal care were 1.7 times more likely to have had a postnatal care visit (OR, 1.67; 95% CI, 1.10–2.54).”[41] Project Mwana, using mobile phones to improve early infant diagnosis of HIV and postnatal follow-up and care, reported that “SMS delivery of results can increase turnaround times by 50% on average, with a greater positive impact in rural facilities.”[42] The Better Border Health Program on the Thailand-Myanmar border combined web-based and mobile technology to generate Antenatal Care/Expanded Program on Immunization (ANC/EPI) visit schedule dates and update scheduling status, showing “ANC/EPI coverage in the study area along the country border improved; numbers of ANC and EPI visits on-time as per schedule significantly increased; there was less delay of antenatal visits and immunizations.” [43] In Uganda, RapidSMS-Maternal and Child Health reported a 27% increase in facility based delivery from 72% to 92% at the end of the twelve months pilot phase. [44] Also, the Rural Extended Services and Care for Ultimate Emergency Relief (RESCUER) Project demonstrated that improved communication and transportation links between traditional birth attendants and health posts resulted in increased and more proximate referrals as well as the improved delivery of healthcare to a large number of pregnant women. A notable impact of the project was that, “the increased number of deliveries under trained personnel and increased referrals to health units led to a reduction of about 50 percent in the maternal mortality rate (MMR) in three years,” [45] although this reduction was not exclusively attributed to the mHealth intervention.

**Figure 1:**
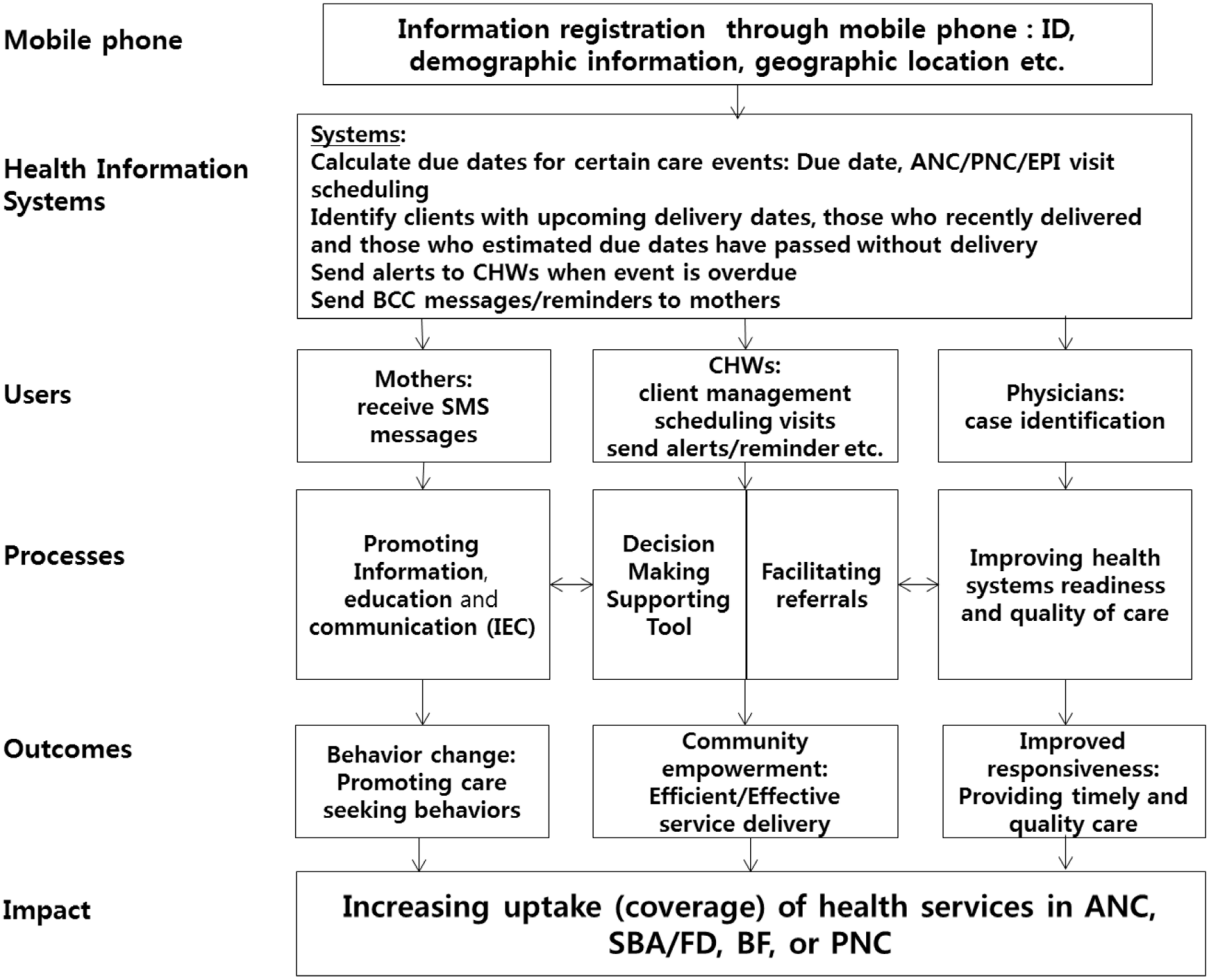
mHealth Health Service Coverage Increase Impact Model. SOURCE: Authors.

The mechanisms by which mHealth promotes service delivery can be explained through an impact logic model. Figure 1 demonstrates how mHealth systems can contribute to increased uptake of health services, including ANC, Skilled Birth Attendance/Facility Delivery (SBA/FD), Breastfeeding (BF), and PNC. The simple yet powerful functions of mobile phones such as data registration/management and texting/calling could facilitate health service delivery in low resource settings by promoting both demand and supply. First, in terms of demand promotion, mHealth can send SMS reminder messages to mothers in order to enhance knowledge and promote timely and appropriate care-seeking and therefore enhance utilization of health services. Second, in terms of supply promotion, mHealth can promote supply management which can reduce the risk of stock-outs and facilitate referrals between CHWs and doctors. [46] mHealth also can enhance performance productivity or efficiency through real-time task monitoring or decision support tools. [38] When appropriate, mHealth could help task shifting through education and training, where limited numbers of skilled health workers are available [47].

Despite these promised results, many projects in developing countries have not established comprehensive quantitative evidence demonstrating the extent to which SMS reminders or calls contribute to improving coverage or changing mortality related to the MNH interventions. Mechael proposed that “the important next step is that key mHealth stakeholders consider focusing on catalyzing the testing and scale up of interventions that show promise in achieving key health outcomes as laid out by the Millennium Development Goals (MDGs) for health.”[48] In the absence of more comprehensive evidence, however, conclusions on priorities and mHealth potential remain merely presumptive.

### What is *LiST*?

The Lives Saved Tool (*LiST,* www.livessavedtool.org) is a software that can be used to identify priority areas for maternal, child and neonatal health investment by allowing users to analyze multiple scenarios to estimate the potential health impact associated with improvements in coverage of interventions of known efficacy. [49]–[54] *LiST,* based in the Spectrum software, was initially developed by the Child Health Epidemiology Reference Group (CHERG) for the WHO and UNICEF with technical content presently managed by the Johns Hopkins Bloomberg School of Public Health’s Institute for International Programs. It combines current mortality rates and health intervention coverage changes over time with evidence of effectiveness to model changes in numbers of maternal, neonatal, child (1–59 month) deaths and stillbirths as well as rates/ratios. Impact can be categorized by (a) year of implementation; (b) population sub-group (e.g. mothers, newborns, children under 5 years); (c) cause of death and/or (d) intervention. [55]–[59] *LiST* marries data on the effectiveness of critical MNH interventions with estimates of service delivery coverage to estimate the impact of implementing a given service/intervention as part of a larger package or as a stand-alone endeavor. Users can modify coverage rates based upon external assumptions about the intensity of support provided (including level and duration of financial support), status of the health infrastructure, quality of program implementation, and other relevant contextual factors. The impact of implementing MNH services as part of a larger continuum of care can then be modeled. [52] Ultimately, a measure of impact emerges in terms of deaths averted. [60] Many studies have demonstrated considerable validity of its modeled results. [50], [52], [53] More specific methods and assumptions behind the *LiST* modeling have been published [56], [61].

### Why *Li*ST

In a recent review of mHealth strategies, [11] we proposed that a reframing of mHealth should be encouraged, viewing mobile technologies as a mechanism to improve the coverage of interventions of known efficacy (e.g. Vaccines, Antenatal Care, Skilled Birth Attendance). This approach facilitates the measurement of the estimated impact of mHealth investments, focusing on coverage and process indicators, instead of distal survival outcomes. In this respect, we explore the use of *LiST* as a tool for planning and prioritizing strategic guidelines for mHealth-based strategies targeting interventions delivered by CHWs, aimed at reducing maternal, newborn and child mortalities. Three prominent features of *LiST* allow it to be a useful modeling tool for estimating the potential effect of mHealth. First, *LiST* uses *coverage* as an input parameter, determining the level of health service coverage increases from the present time toward a target year, to model the mortality impact of scaling up a specific maternal, newborn and child health intervention. As noted above, given the nature of mHealth facilitating health service provision of proven intervention, coverage–defined as a percent of a population in need who received a particular health intervention–is often regarded as an important health service outcome indicator in mHealth program evaluation, reflecting measures of access and quality of health provision. Second, *LiST* models the effects of changes in *individual-level interventions* such as exclusive breastfeeding of a newborn or Oral Rehydration Solution (ORS) treatment of a child with diarrhea. This is applicable to the characteristics and benefits of mHealth with its capacity in connecting and linking health services for individuals. Third, *LiST* modeling can be used as a tool to investigate not only single interventions, but also a set of specific interventions, which may be *combined into packages.* This approach can mimic mHealth programming, which often promotes an integrated approach across different health interventions through a common service delivery platform. For example, text messaging can be used to distribute various behavior change messages, reminders of services for ANC visits, breastfeeding promotion, and vaccination scheduling. More importantly, *LiST* systematically calculates the mortality impact of individual or combined interventions based on the evidence pool embedded into the system. This cross-cutting approach can not only improve the potential value of mHealth investments, but could create synergy and improve efficiency in implementing several interventions at the same time. Rather than creates new silos of health innovation, mHealth optimizes a standardized service delivery protocol to connect resources across health system programs. In this respect, this study explores the use of *LiST* to model mHealth strategies in two countries, Bangladesh and Uganda, estimating the neonatal mortality averted from different scenarios involving maternal and newborn health program interventions.

## Methods

Bangladesh and Uganda were selected in particular due to their relatively high neonatal mortalities (27 and 26 per 1,000 live births respectively, as of 2010) and the fact that mHealth programs have been positively recognized by their governments and actively implemented on the ground. The baseline data sources, embedded in *LiST*, draw from multiple academic publications and statistics from Demographic and Health Surveys (DHS), Multiple Indicator Cluster Survey (MICS), UNICEF, and the World Bank. Among the inputs in *LiST*, four key interventions–Antenatal care (ANC), Skilled birth attendance and/or Facility delivery (SBA/FD), Breastfeeding promotion (BF), and Postnatal care (PNC) –were identified as representative core health services across the continuum of care for MNH. Specific definitions and the components of the interventions applied in the *LiST* are explained in Table 2 and File S2. Using 2015 as the target year, scenarios were created examining various coverage increase targets and the impacts of bundling packages of interventions.

**Table 2:** *LiST* Interventions and Coverage Increase Scenarios in Bangladesh and Uganda (input parameters of baseline year in 2011 and target year in 2015).

*LiST* Interventions (selected)			Bangladesh				Uganda			
			Baseline (2011)	Projected coverage increase (2015)			Baseline (2011)	Projected coverage increase (2015)		
				10%	30%	50%		10%	30%	50%
Pregnancy	Antenatal care (ANC 4+)		25.5	28.1	33.2	38.3	47.6	52.4	61.9	71.4
Childbirth	Skilled birth attendance*		31.7	34.8	41.2	47.6	58.0	63.8	75.4	87.0
	Facility delivery* (Clinic and Hospital)		28.8	31.7	37.4	43.2	57.4	63.1	74.6	86.1
	Home deliveries** (% of all deliveries)	Unassisted deliveries**	68.3	65.2	58.8	52.4	42.0	36.2	24.6	13.0
		Assisted deliveriesat home**	2.9	3.1	3.8	4.4	0.6	0.7	0.8	0.9
	Facility deliveries**(% of all deliveries)	Essential care**	25.9	15.8	18.7	21.6	14.3	15.8	18.6	21.5
		BEmOC**	0.0	9.5	11.2	13	8.6	9.5	11.2	12.9
		CEmOC**	2.9	6.3	7.5	8.6	34.4	37.9	44.8	51.7
Breastfeedingpromotion and prevalence(<1 month)	Promotion of breastfeeding		36.3	39.9	47.2	54.5	34.8	38.3	45.2	52.2
	Exclusive breastfeeding**		84.5	84.9	85.6	86.3	89.9	90.1	90.6	91.0
	Predominant breastfeeding**		5.9	5.7	5.5	5.2	5.0	4.9	4.7	4.4
	Partial breastfeeding**		9.6	9.4	9.0	8.5	5.1	5.0	4.8	4.5
Preventive	Preventive postnatal care(Thermal care, Clean postnatal practice)		29.6	32.6	38.5	44.4	2.8	3.1	3.6	4.2

SOURCE: *LiST.*

Notes: For detailed definitions and data sources, see *LiST* manuals and published articles [56], [61] [67];baseline coverage data were compiled from Demographic and Health Surveys (DHS: Uganda and Bangladesh, 2011); Multiple Indicator Cluster Survey (MICS Round 3: Bangladesh, 2006).

*Coverage measure of SBA includes coverage measure of FD. Thus we modeled coverage increase for SBA and FD simultaneously as 10%, 30%, and 50%. Data course of SBA and FD is from DHS/MICS and percentages of home deliveries and facility deliveries are based on *LiST* imbedded algorithms.

**Estimations of home deliveries (unassisted deliveries, assisted deliveries at home), facility deliveries (essential care, BEmOC, CEmOC), exclusive breastfeeding, predominant breastfeeding, and partial breastfeeding are derived from the *LiST* imbedded algorithms.

**Antenatal care (ANC 4+):** Percent of pregnant women with at least 4 antenatal care visits during their pregnancy. The intervention includes Routine (TT, IPTp, Syphilis detection and treatment), Nutritional (Calcium supplementation), Case management (Diabetes, Management of pre-eclampsia), Other (Fetal growth restriction detection and management). This analysis does not include iron-folic acid. Data source of ANC is from DHS/MICS.

**Skilled Birth Attendance (SBA):** Percent of children born who are attended by a skilled attendance, including doctors, nurses, midwives- in a facility or home. An SBA in the home is defined as a skilled birth attendant who deliveries the infant at home without benefit of referral to a facility in case of emergency. An SBA in a facility is defined as a medically skilled attendant who has the ability and facilities needed to monitor labor progress with a partograph and detect complications. Episiotomy is available, if needed. Infection control is covered under clean birth practices; **Facility delivery (FD):** Percent of children born in an institution. Unassisted deliveries: Percent of deliveries without skilled attendance in the home.

- Assisted deliveries at home: Percent of deliveries with a skilled attendant in the home.

- Essential care: Percent of deliveries including monitoring labor progress with a partograph, detection of complications and infection control via a clean delivery, Episiotomy is available, if needed. For the neonate, this includes routine care practices including: immediate drying, skin-to-skin contact or immediate wrapping for thermal care and clean cord cutting.

- BEmOC (Basic Emergency Obstetric Care): Percent of deliveries at a health center and covers case management of direct obstetric complications. The intervention includes: Case management of ante-partum hemorrhage, prolonged/obstructed labor, post-partum hemorrhage and severe infection. Methods include: shock management, pain relief, ABC, IV fluids, instrumental delivery and manual removal of the placenta and retained products.

- CEmOC (Comprehensive Emergency Obstetric Care): Percent of deliveries at a hospital and covers case management of direct obstetric complications. In addition to including all interventions in Basic Emergency Obstetric Care, additional methods include: ultrasound, culdocentesis, induction, laparotomy, salpingectomy, blood transfusion, caesarian section, hysterectomy, symphisiotomy, balloon tamponade, uterine ligature, MRVOP, surgical infection control and episiotomy.

- Users can change any of the assumptions in the *LiST* by simply uncheck the box ‘Allow *LiST* to calculate place and level of delivery’ to allow direct entry of these values. The sum of all five delivery levels must be no more than 100%. Note that the values listed assume the highest level of care that is available for that particular delivery. So the percent of deliveries with essential care selected assume that none of these pregnancies have BEmOC or CEmOC available.

**Exclusive breastfeeding (BF):** Percent of children 0–11 months receiving only breast milk for food (plus medication, vaccines, and vitamins).

**Preventative postnatal care (PNC):** Percent of infants with a postnatal health contact/visit within 2 days of birth.

Three analyses were conducted for each of the countries to determine the estimated impact of different intervention packages on neonatal mortality. The first analysis modeled improving coverage of four key MNH interventions, ANC, SBA/FD, BF and PNC to three different target coverage levels–10%, 30%, and 50%. The target coverage in 2015 was defined in a *relative* manner by multiplying the baseline coverage of each intervention in 2011 by 110%, 130% and 150%. We do not have enough evidence to set particular *absolute* target coverage numbers for any intervention. These hypothetical coverage increase scenarios can be considered as a fairly feasible and conservative approach, given the practices and evidence, as discussed above. We assumed a linear growth in coverage, from the baseline year 2011 to the target year 2015. The mortality impact outcomes were compared across different coverage increase scenarios, and health interventions scenarios. (Table 2).

The second analysis focused on bundled packages of interventions based on common mHealth strategies, as mHealth strategies can facilitate integrated interventions through a common communication channel or service delivery pathway. For this analysis, we compared the impact of increasing coverage of combined interventions (“BF & PNC” and “ANC, SBA/FD, BF & PNC”, called All-combined) at the same target coverage levels (10%, 30% and 50%, respectively). The bundling of key MNH interventions centered on data collection/registration and SMS reminder for scheduled visits (ANC), referral calling and emergency medical referral (SBA/FD), and healthy behavior promotion via text messages (BF & PNC).

The final analysis was created to identify the optimal mix of services and tradeoffs in coverage by comparing four individual and two bundled interventions scenarios: 1) ANC, 2) SBA/FD, 3) BF, 4) PNC, 5) BF & PNC, and 6) All-combined. While vaccination is one of the key interventions in the MNH continuum of care, it was not included in these analyses due to the already high baseline coverage levels reported for both countries, 86% in Uganda and 95% in Bangladesh in 2011.

## Results

**Figure 2:**
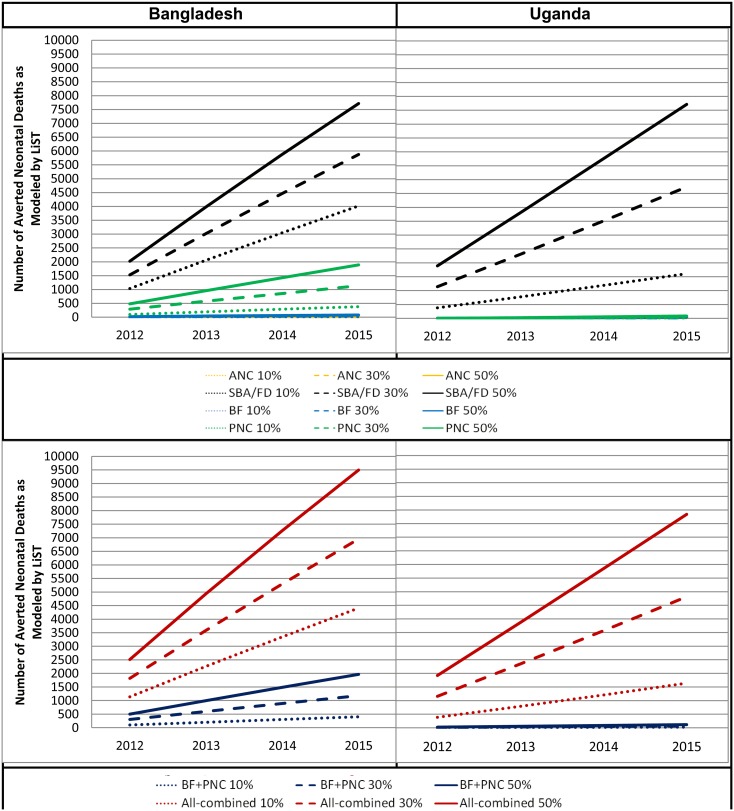
Neonatal Mortality Impacts by Various MNH Interventions and Coverage Scenarios in Bangladesh and Uganda in 2015. SOURCE: *LiST.*

Coverage values modeled for Bangladesh and Uganda for each selected intervention at target year 2015 are presented in Table 2. Modeled numbers of neonatal deaths averted in 2015 are presented in Table 3. Comparisons of the mortality impact of the four individual and two bundled interventions scenarios at all three coverage levels are presented by country in Figure 2.

**Table 3:** Neonatal Mortality Impacts by Various MNH Interventions and Coverage Scenarios in Bangladesh and Uganda in 2015.

Interventions	Illustrative mHealth Strategies	Coverage increaseby 2015	Projected number of neonatal lives saved							
			Bangladesh				Uganda			
			2012	2013	2014	2015	2012	2013	2014	2015
ANC	Data collection and management(e.g. Risk assessment and classification,Vital events tracking, adherence reminder);SMS texting for health promotion andscheduled visits reminder	10%	0	0	0	0 (1)***	1	2	3	5 (0.8)
		30%	0	1	1	1 (1)	3	7	10	14 (0.79)
		50%	0	1	2	2 (1)	5	11	17	23 (0.78)
SBA/FD	Emergency medical referral(e.g. referral calling)	10%	1038	2055	3048	4016 (0.74)	381	776	1187	1611 (0.76)
		30%	1530	3021	4470	5877 (0.74)	1141	2312	3512	4738 (0.76)
		50%	2021	3984	5882	7717 (0.74)	1892	3811	5753	7714 (0.75)
BF	SMS texting for health promotion	10%	4	8	12	16 (0.75)	2	3	5	7 (0.71)
		30%	12	24	36	48 (0.75)	5	8	14	18 (0.72)
		50%	20	41	61	80 (0.75)	8	16	26	35 (0.77)
PNC	SMS texting for health promotion(and scheduled visits reminder)	10%	98	194	289	383 (0.74)	0	6	12	17 (1)
		30%	290	576	858	1135 (0.74)	0	15	30	47 (1)
		50%	482	958	1427	1888 (0.74)	0	26	54	83 (1)
BF & PNC	SMS texting for health promotion(and scheduled visits reminder)	10%	102	202	301	399 (0.74)	5	11	18	24 (0.79)
		30%	302	600	894	1183 (0.74)	16	32	50	68 (0.76)
		50%	502	999	1487	1968 (0.74)	27	56	86	118 (0.77)
All-combined: ANC, SBA/FD, BF &PNC	Data collection and management(e.g. Risk assessment and classification, Vital events tracking, adherence reminder);SMS texting for health promotion and scheduled visits reminder;Emergency medical referral (e.g. referral calling)	10%	1141	2258	3346	4405 (0.74)	388	790	1208	1639 (0.76)
		30%	1820	3587	5298	6951 (0.74)	1160	2349	3569	4814 (0.76)
		50%	2512	4934	7262	9496 (0.74)	1924	3874	5847	7839 (0.75)

SOURCE: *LiST.*

Note: ()*** is a percentage change in NMR from baseline in 2011 to 2015.

Comparing single interventions, SBA/FD provided the biggest mortality impact relative to other intervention scenarios with PNC, BF, and ANC, across both countries. For example, at 50% coverage target scenario, SBA/FD was estimated to save 7717 neonates in Bangladesh and 7714 neonates in Uganda. Next, PNC was estimated as the second biggest mortality impact in Bangladesh with 1888 lives saved, while only 83 lives saved in Uganda. In fact, PNC lead to fairly limited mortality reduction impact in Uganda because the baseline coverage was very low as only 2.8%, compared to 29.6% for Bangladesh. BF promotion also lead to fairly limited impact to both countries as 80 lives saved in Bangladesh with and 35 lives saved in Uganda. Examining the ANC scenario, the least mortality impact was estimated to both countries with 23 saved lives in Uganda and only 2 saved lives of neonates in Bangladesh.

For the bundled interventions, All-combined interventions at 50% coverage scenario demonstrated, not surprisingly, the largest impact in terms of neonatal mortality; the number of neonatal deaths averted was higher for Bangladesh with 9496 saved lives than 7839 in Uganda. Next, the bundled packages of BF & PNC (with 50% coverage scenario) produced considerable impact with 1968 lives saved in Bangladesh, while only 118 lives saved in Uganda. The difference is mainly driven by the considerable mortality impact from PNC intervention in Bangladesh compared to Uganda. However, overall it is important to consider that initial baseline coverage of each intervention and mortality level differed by country.

## Discussion

Given that SBA/FD emerged as the most effective intervention in terms of mortality impact in both countries, a possible recommendation based on this modeling experiment would be for a strategic focus on childbirth related interventions. Accordingly, this analysis supports the implementation of mHealth strategies such as facilitating emergency medical referrals for childbirth-associated interventions during intrapartum crises. The findings further highlight that despite a low baseline coverage of SBA/FD in Bangladesh in 2011–almost two times lower (31.7%) than (58%) in Uganda–Bangladesh demonstrates considerable potential to reduce neonatal mortality by focusing on SBA/FD interventions or combining SBA/FD with other interventions into bundled packages, at the 50% increased coverage level. Further, given the relatively greater mortality impact with bundled packages of BF & PNC in Bangladesh, one cross-cutting recommendation would be to use SMS text messaging to encourage health promotion and behavior change, leveraging the benefits of combined interventions, focusing on Information, Education, and Communication (IEC) strategies. With the ability to promote health service delivery, mHealth interventions may offer the opportunity to increase coverage more effectively and rapidly than standard methods.

In this early application of *LiST* on modeling the potential impact of mHealth strategies, it is also important to note some similarities and distinctions between general assumptions of *LiST* based on standard healthcare delivery and the unique characteristics of mHealth strategies. In addition, careful consideration of major limitations and caveats in this approach is necessary to correctly interpret the mortality impact and suggest appropriate conclusions from the findings.

First, the level of mortality impact is influenced by the reported initial baseline coverage level and evidence from standard care practices. [File S2] For example, the baseline PNC coverage is only 2.8% in Uganda and therefore the target coverage was set as 4.2% in the case of a 50% coverage increase. Accordingly, the modeling resulted in a limited mortality reduction as well. In case of BF promotion (with 50% increase scenario), the *LiST* modeling algorithm assigned a mere 1–2% increase in BF prevalence of exclusive breastfeeding based on the embedded country specific evidence in Bangladesh and Uganda. mHealth interventions, however, could potentially enhance BF prevalence significantly through individualized, timely, and targeted interventions for behavior change, compared to a standard BF promotion intervention. On the other hand, in case of SBA/FD delivery (with 50% increase scenario), the coverage projection of CEmOC in Uganda is estimated as 86% by 2015, based on the baseline coverage as 51.7% in 2011. mHealth referrals alone, however, does not guarantee such a high coverage uptake without well-prepared and organized health systems including transportation access, medical commodities/equipment and skilled health professionals.

Despite these caveats, this analysis adopted a relative target coverage approach, evenly distributed across each year, instead of setting an absolute target coverage number for 2015 or allowing for a fixed incremental coverage increase in each year through 2015. As current mHealth studies have seldom presented information pertaining to targets set for coverage nor measured coverage over time precisely, it was challenging to base these growth projections on actual field-based performance. Yet, the relative target coverage modeling approach, using the existing modeling algorithms based on standard care practices, allows for a more consistent and conservative estimation across various interventions and combinations of interventions. Here, we assumed that the baseline coverage status reflects the level of the health system capacity in scaling up a given intervention and thus can be used to determine approximate target coverage for 2015.

Second, like many other modeling tools, the analysis (and *LiST*) does not systematically consider health systems constraints in achieving the target coverage. Even for well-known effective interventions, the process of making real impact involves understanding the substantial constraints or obstacles within the broader context of the health systems. On one hand, mHealth strategies may leverage economies of scale and thus increase the coverage in some exponential fashion, as experience and efficiency with the system improve. On the other hand, they are more likely involve dis-economies of scale at the same time and increase marginal costs when the intervention faces a number of health systems constraints and challenges in remote areas or resource limited settings. For simplicity, our analyses assume linear trends over time, but acknowledge that complex health systems and bottlenecks in expanding programs are likely to lead to a non-linear adoption in intervention coverage.

Accordingly, the analysis (and *LiST*) does not explain *how* the target coverage can be achieved. As noted, considering the multifaceted complexity and logistic challenges to scaling up health services,[62].

mHealth strategies, like any health system strengthening innovation, requires well-trained health care providers, sound supply chain management and stable technological platforms which are often critical challenges in many resource-limited settings. [63] As discussed earlier, while useful functions of mHealth are expected to overcome health systems gaps and obstacles, in reality, the success of well-functioning mHealth system is significantly dependent upon the level of capacity and readiness of the given health system. For instance, while our findings present the considerable impact on SBA/FD, if SBA/FD is significantly constraint by geographical inaccessibility or lack of skilled health professionals, then mHealth is likely to have limited impact. Alternatively, if SBA/FD is available, accessible and affordable but under-utilized, then mHealth may have a significant role in increasing the coverage. In this respect, some successful mHealth strategies may incorporate additional incentive mechanisms (ex. conditional cash transfer, voucher programs) or access mobilization strategies (ex. ambulance services) together to promote care-seeking practice or facility delivery for pregnant women living in remote areas [34]–[36], [64].

Third, this analysis does not consider *effective coverage* [65]– comprehensive sub-components of a given intervention package. While *LiST* allows the user to control coverage inputs for sub-components of some service interventions, this modeling did not incorporate specific performance scenarios of sub-components. For example, under ANC, there are several subcomponents such as tetanus toxoid vaccination (TT), intermittent preventive treatment of pregnant women for malaria (IPTp), calcium supplementation, multiple micronutrient supplementation, hypertension disease management, diabetes case management, malaria case management, and management of pre-eclampsia (MgSO4). In this modeling study, we used a basic indicator of ANC–defined as “percent of pregnant women who go to 4 or more antenatal care visits during the pregnancy (ANC4+)”–with automatically calculated components based on standard assumptions from the current status of practice in the country. However, further mortality reductions would likely be observed if mHealth strategies, such as e-IMCI, delivering improvements in ANC coverage were also accompanied by improvements in the quality and comprehensiveness of the necessary sub-components of that service. In this respect, the *LiST* results provide a conservative estimate of the potential impact of mHealth strategies in this study. In other words, future studies may develop scenarios that illustrate how effective coverage-based models are expected to deliver differences in mortality reduction associated with an intervention, either crudely, or by accounting for specific sub-components of the intervention, as needed.

Beyond modeling, prospective impact evaluation research would be useful to validate these models and provide more informed projections on potential coverage change associated with various mHealth solutions across different contexts over time. For instance, prospectively obtained data on coverage could also be manually added to a *LiST* model, allowing estimates of associated likely mortality reductions. Notably, this approach highlights the potential of coverage indicators to serve as the primary metrics of an evaluation strategy. Further, when possible, for even more precise estimations for a specific program, the program may also try to obtain available regional data on measures of health status (e.g. levels of risk factors and population exposures, baseline cause-specific mortality estimates etc.) to modify *LiST* modeling inputs. These indicators are often a less expensive (and time-consuming) endeavor to measure than the mortality endpoints of impact studies, yet can serve well for decision-makers seeking to understand the potential – or actual – health benefit of different investments into mHealth strategies.

In summary, with a special focus on coverage as a primary measure of mHealth impact, *LiST* allows incorporation of two potentially advantageous aspects of mHealth: accelerated coverage “uptake” and improved coverage “quality”. For example, in terms of the uptake, if an intervention (not limited to mHealth) protocol and sub-components are similar to the defined intervention criteria in the *LiST*, different coverage increase rate among various interventions can be used to compare the mortality impact. In terms of the quality, if an intervention promotes comprehensiveness or compliance of service provision of an intervention package, users can control some available sub-components in the *LiST* to estimate the different mortality impact. In addition, when appropriate, programs may set certain target coverage based on relevant evidence from policy goals or resource constraints–instead of relative target scenarios based on baseline coverage–to estimate potential mortality impact. In this way, *LiST* can promote a greater allocative efficiency in a societal perspective, to evaluate performance of health service provision across various interventions, using coverage indicators. Further, this functional merit allows itself to be a benchmarking tool by examining different impact estimates across various types of intervention strategies or between estimated target impact and observed real impact. The gaps may allow researchers and implementers to infer potential bottlenecks in the given health system or discuss priority intervention strategies.

Finally, while country-level impact estimation from *LiST* modeling scenarios may be extremely useful in setting priorities for mHealth (and even conventional public health) strategies, it is important to ensure that the findings serve as ingredients for informed discussion-making and not drive the selection of strategies in a vacuum. Although our findings suggest the most effective intervention to be SBA/FD coverage improvements, based on the mortality impact estimations, *LiST* does not account for the cost implications of increases in coverage or other health systems constraints. As noted, mHealth solutions often require additional funding and efforts to establish or connect to existing back-end health information systems, equip staff with mobile devices, conduct research to ensure effective deployment, advocacy activities to raise awareness about the program and improvements, and mechanisms to support and supervise health workers. [66] Moreover, various cultural, social, and political contextual factors mediate adoption and adaptation of new mHealth systems. Thus, decisions on where to focus resources and investments will have to be determined after taking into consideration the feasibilities of interventions and the existing health systems’ strengths and limitations in any particular context.

## Conclusions

This analysis explored an application of the *LiST* tool for planning and prioritization of mHealth strategies which could improve coverage in four key MNH intervention areas: ANC, SBA/FD, BF promotion, and PNC at three different target coverage level improvements–10%, 30%, and 50%. Models were run to estimate and compare the mortality impact of four key individual and two combined MNH interventions in the two countries examined, Bangladesh and Uganda. The model clearly identifies the potential expected benefits associated with some strategies and not others, while establishing possible ‘aspirational’ targets for projects in these focal areas. In the context of Bangladesh and Uganda, the greatest benefits would likely be associated with mHealth strategies targeting MNH interventions at childbirth facilitating SBA/FD services. Moreover, Bangladesh would see the positive benefit through a bundled interventions approach leveraging a combination of BF promotion & PNC.

Although further validation of this model is needed, this study represents to our knowledge, the first use of *LiST* as a strategy to identify, for innovators and governments, priority areas for mHealth strategic focus and investment. Our study may not only serve to improve the scope and quality of the assumptions in *LiST*, but also highlight areas where further research is needed on effectiveness of interventions. Many of the interventions, where insufficient data exist to estimate impact, are being used and/or promoted in countries. Clearly if better health policy decisions are to be made, additional efforts to collect data on the efficacy and effectiveness of these interventions need to be prioritized.

We have described how the *LiST* tool would also allow existing projects in these domains to review their progress in terms of intervention coverage. This would enable projects to set benchmarks and monitor progress against projected targets, while providing information derived from rigorous, efficacy-trial driven expectations. The importance of mHealth scale-up has been broadly emphasized in the mHealth community, but it is necessary to guide such scale up efforts and investment in ways that are most likely to achieve the mortality reduction targets set by national goals and global calls to action such as the MDGs, not merely to expand programs. The results of this analysis can contribute to discussions about how such plans can be formulated, emphasizing the likely highest-impact interventions for prioritization, considering the unique potential for synergy across multiple areas that mHealth solutions typically allow. As the deadline for achieving the MDGs quickly approaches, use of available tools such as *LiST* can help us further leverage the benefit of mHealth by articulating the most appropriate delivery points in the continuum of care to save lives.

## Supporting Information

File S1 **Systematic Review Protocol on mHealth for Community Health Workers on Maternal and Newborn Health Service Delivery in Low and Middle Income Countries.**
(DOCX)

File S2 ***LiST*** **Data Sources for the Modeling Analyses.**
(XLSX)
