# Correction: Using the Lives Saved Tool (*LiST*) to Model mHealth Impact on Neonatal Survival in Resource-Limited Settings

**DOI:** 10.1371/journal.pone.0106980

**Published:** 2014-08-27

**Authors:** The PLOS ONE Staff

There is an error in the third to last sentence of the third paragraph in the mHealth for community health works on maternal and newborn health services section of the Introduction. The correct sentence is: In Rwanda, RapidSMS-Maternal and Child Health reported a 27% increase in facility based delivery from 72% to 92% at the end of the twelve months pilot phase.

There is an error in Table 1. The country in the RapidSMS-MCH row should be Rwanda, not Uganda. Please see the corrected Table 1 here.

**Table 1:** Examples of mHealth programs on MNH services through CHWs**.**

Project	Country	Organization	Interventions	mHealth strategies	mHealth benefit/impact evidence on service provision
Wired Mothers	Tanzania	Danish International Development Cooperation, University of Copenhagen	(i) Family planning (ii) Behavior changes through Information, Education and Communication (IEC) (iii) Antenatal care(ANC)/Expanded Program on Immunization (EPI)/Postnatal care(PNC) (iv) Skilled birth attendance(SBA)/Facility delivery (FD)	(i) Data collection and management (e.g. Risk assessment and classification, Vital events tracking, adherence reminder) (ii) SMS texting for health promotion and scheduled visits reminder (with mobile phone voucher components)	“The mobile phone intervention was associated with an increase in antenatal care attendance. In the intervention group 44% of the women received four or more antenatal care visits versus 31% in the control group (odds ratio (OR), 2.39; 95% confidence interval (CI), 1.03-5.55). There was a trend towards improved timing and quality of antenatal care services across all secondary outcome measures although not statistically significant.” [22]
					“The mobile phone intervention was associated with an increase in skilled delivery attendance: 60% of the women in the intervention group versus 47% in the control group delivered with skilled attendance. The intervention produced a significant increase in skilled delivery attendance amongst urban women (OR, 5.73; 95% CI, 1.51–21.81), but did not reach rural women.” [34]
					“The perinatal mortality rate was lower in the intervention clusters, 19 per 1000 births, than in the control clusters, 36 per 1000 births. The intervention was associated with a significant reduction in perinatal mortality with an OR of 0.50 (95% CI 0.27-0.93). Other secondary outcomes showed an insignificant reduction in stillbirth (OR 0.65, 95% CI 0.34-1.24) and an insignificant reduction in death within the first 42 days of life (OR 0.79, 95% CI 0.36-1.74).” [40]
Maternal and Newborn Health in Ethiopia Partnership (MaNHEP)	Ethiopia	University Research Co., LLC, Quality Improvement Advisor for the Maternal and Newborn Health in Ethiopia Partnership	(i) Family planning (ii) Behavior changes through IEC (iii) ANC/EPI/PNC	(i) SMS texting for health promotion and scheduled visits reminder (e.g. promotion of community maternal and newborn health family meetings and labor and birth notification)	“Women who had additionally attended 2 or more CMNH meetings with family members and had access to a health extension worker’s mobile phone number were 4.9 times more likely to have received postnatal care (OR, 4.86; 95% CI, 2.67-8.86; *P* _.001).” [41] “Notification of health extension workers for labor and birth within 48 hours was closely linked with receipt of postnatal care. Women with any antenatal care were 1.7 times more likely to have had a postnatal care visit (OR, 1.67; 95%; 95% CI 1.10-2.54; *P* _ .001).” [41]
E-IMCI	Tanzania	D-Tree	(i) ANC/EPI/PNC (ii) Behavior changes through IEC	(i) Point of care decision support through compliance to IMCI protocols	“For all ten critical IMCI items included in both systems, adherence to the protocol was greater for eIMCI than for pIMCI. The proportion assessed under pIMCI ranged from 61% to 98% compared to 92% to 100% under eIMCI (p < 0.05 for each of the ten assessment items).” [25]
Project Mwana	Zambia, Malawi	UNICEF	(i) HIV-antiretroviral therapy (ART) surveillance and treatment	(i) Data collection and management (ii) SMS texting for health promotion and scheduled visits reminder	“ SMS delivery of results can increase turnaround times by 50% on average, with a greater positive impact in rural facilities” [42]
Better Border Healthcare Program	Thailand-Burma	Mahidol University, Thailand	(i) Family planning(ii) ANC/EPI/PNC	(i) Data collection and management (ii) SMS texting for health promotion and scheduled visits reminder	“ANC/EPI coverage in the study area along the country border improved; numbers of ANC and EPI visits on-time as per schedule significantly increased; there was less delay of antenatal visits and immunizations” [43]
RapidSMS-MCH	Rwanda	Ministry of Health Uganda, UNICEF	(i) Family planning (ii) Behavior changes through IEC (iii) ANC/EPI/PNC (iv) SBA/FD	(i) Data collection and management (ii) SMS texting for health promotion and scheduled visits reminder	Study reported “a 27% increase in facility based delivery from 72% twelve months before to 92% at the end of the twelve months pilot phase.” [44]
Rural Extended Services and Care for Ultimate Emergency Relief (RESCUER)	Uganda	Ministry of Health, UN Population Fund and the Uganda Population Secretariat	(i) Behavior changes through IEC (ii) ANC/EPI/PNC (iii) -SBA/FD	(i) Emergency medical referral (e.g. referral calling) with transportation services	“improved communication and transportation links between the Traditional Birth Attendants (TBAs) and the health posts resulted in increased and more timely referrals as well as the improved delivery of healthcare to a large number of pregnant women”… “The increased number of deliveries under trained personnel and increased referrals to health units led to a reduction of about 50 percent in the maternal mortality rate (MMR) in three years” [45]
M4RH	Kenya, Tanzania	USAID, FHI 360’s PROGRESS (Program Research for Strengthening Services)	(i) Family planning (ii) Behavior changes through IEC	(i) Data collection and management (ii) SMS texting for health promotion and scheduled visits reminder	User interviews reported various positive responses including “the text messaging service was perceived as being private, convenient, and cost-effective.” [33]
PREVEN	Peru	Cell-Preven	(i) Sexual and reproductive health surveillance and service delivery	(i) Data collection and management (ii) SMS texting for health promotion and scheduled visits reminder	Lessons include “Two-way information systems are more than just collecting data. They provide feedback and support to health care workers in the field. Many times, only managers have information that allows them to monitor and evaluate data but these systems do not prove any aggregate value to health care workers in the field. A well-designed information system has to support and enhance the performance of all user levels in a secure environment.” [30] “Prahalad (2005) has reported that health workers in some developing countries spend as much as 40% of their time filling out forms, compiling and copying data from different pro-grams (e.g., tuberculosis, malaria, HIV/AIDS, etc.). By choosing the most appropriate information technology, we can avoid duplication and deploy different devices—i.e., cell phones, Internet—to report from each public health program.” [30]
Aceh Besar Midwives	Indonesia	UNICEF, UNFPA, and World Vision	(i) Behavior changes through IEC (ii) ANC/EPI/PNC (iii) SBA/FD	(i) Data collection and management (ii) SMS texting for health promotion and scheduled visits reminder (iii) Emergency medical referral (e.g. referral calling)	“Findings from the project indicate that the mobile phone has proven to be an effective and efficient device for facilitating smoother communication, and allowing speedier emergency response. The system also aids in gathering and disseminating health-related information to midwives, who in turn convey this knowledge to the patient community.” [13]
MAMA	Bangladesh, India, and South Africa	mHealth Alliance	(i) Family planning (ii) Behavior changes through IEC	(i) Data collection and management (ii) SMS texting for health promotion and scheduled visits reminder	MAMA Bangladesh Aponjon project represented “a 37% increase over a 2011 national baseline of 26% attending four ANC visits. It is also important to note that 45% of the Aponjon subscribers went to a facility for delivery and 32% chose safe delivery at home” [32]
MOTECH	Ghana	Grameen Foundation	(i) Family planning (ii) Behavior changes through IEC (iii) ANC/EPI/PNC (iv) SBA/FD	(i) Data collection and management (ii) SMS texting for health promotion and scheduled visits reminder (iii) Emergency medical referral (e.g. referral calling)	Comprehensive observational studies demonstrated lessons learned and key future implications. [28] Evaluation is on-going with Grameen Foundation, Healthcare Innovation Technology LAB (HITLAB), and Ghana’s School of Public Health.[29]
