# Supplementary material for: Using the Lives Saved Tool (LiST) to Model mHealth Impact on Neonatal Survival in Resource-Limited Settings
Source: PLoS One. 2014 Jul 11;9(7):e102224. doi: 10.1371/journal.pone.0102224 (PMC4094557; doi:10.1371/journal.pone.0102224)
Supplement: File S1 — Systematic Review Protocol on mHealth for Community Health Workers on Maternal and Newborn Health Service Delivery in Low and Middle Income Countries. (DOCX) [file pone.0102224.s001.docx]

**File S1.**

Caption/Headline: Systematic Review Protocol on mHealth for Community Health Workers on Maternal and Newborn Health Service Delivery in Low and Middle Income Countries

Source: Authors

Using the search engines, PuBMED, EMBASE, and SCOPUS, systematic literature review was conducted to identify relevant articles on mHealth for community health workers on reproductive, maternal and newborn child health services in low and middle income countries. Literatures were initially collected based on publication dates from January 1, 2000 to April 20, 2014 and language in English. (n indicates number of identified literature)

**
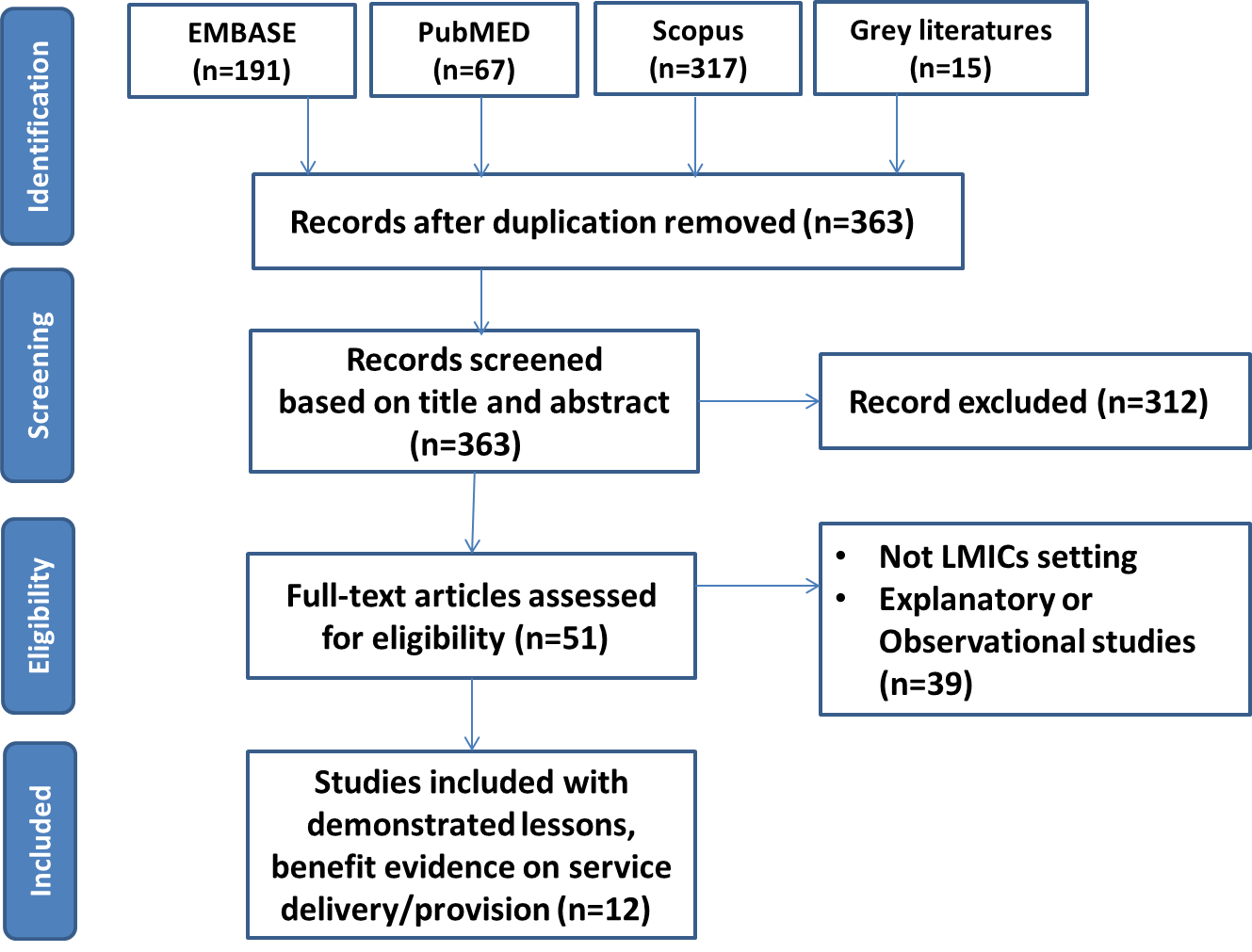
**

| **PubMed** | N=67 |
| --- | --- |
| **MNCH** | "Maternal Health Services"[Mesh] OR "Infant, Newborn"[Mesh] OR “Family Planning Services” [Mesh] OR “reproductive health”[tiab] OR “newborn”[tiab] OR “adolescence”[tiab] OR “pregnancy”[tiab] OR “birth”[tiab] OR “child birth”[tiab] OR “obstetric care”[tiab] OR “postpartum”[tiab] OR “postnatal”[tiab] OR "Neonate"[tiab] OR "Neonates"[tiab] OR "infant"[tiab] OR "infants"[tiab] OR “Child Health Services”[Mesh] OR “Child Health”[tiab] OR “IMCI”[tiab] OR “eIMCI”[tiab] OR “e-IMCI”[tiab] |
| **mHealth** | "Health Information Management"[Mesh] OR "Health Information Systems"[Mesh] OR "Health Communication"[Mesh] OR "Mobile technology"[tiab] OR “Mobile technologies” [tiab] OR "mHealth"[tiab] OR “m-Health”[tiab] OR "SMS"[tiab] OR "short message service"[tiab] OR "text messages"[tiab] OR "texting"[tiab] OR "mobile phone"[tiab] OR “mobile phone technologies” [tiab] OR “mobile phone technology”[tiab] OR “wireless” [tiab] Or “wireless technologies” [tiab] OR “wireless technology”[tiab] OR “cellular phone”[tiab] OR “call”[tiab] OR “calling” [tiab] OR “PDA”[tiab] |
| **CHW** | "Community Health Workers"[Mesh] OR “CHWs”[tiab] OR “Community Health Worker” [tiab] OR "Village Health Worker"[tiab] OR "Village Health Workers"[tiab] OR “Frontline Health Workers”[tiab] OR “midwives”[tiab] OR “health workers”[tiab] |
| **SCOPUS** | N= 317 |
| **MNCH** | TITLE-ABS-KEY (“Maternal Health" OR “Maternal Care” OR "newborn" OR “newborns” OR "Family planning services" OR “adolescence” OR “pregnancy” OR “birth” OR “child birth” OR “obstetric care” OR “postpartum” OR “postnatal” OR "Neonate" OR "Neonates" OR "infant" OR "infants" OR “Child Health Services” OR “Child Health” OR “IMCI” OR “eIMCI” OR “e-IMCI”) |
| **mHealth** | TITLE-ABS-KEY ("Mobile technology" OR “Mobile technologies” OR "mHealth" OR “m-Health” OR "short message service" OR “short message services” OR "text messages" OR "texting" OR "mobile phone" OR “mobile phone technologies” OR “mobile phone technology” OR “wireless” Or “wireless technologies” OR “wireless technology” OR “cellular phone” OR “call” OR “calling” OR “PDA”) |
| **CHW** | TITLE-ABS-KEY (“community care" OR “Community Health Workers” OR "CHW" OR "CHWs" OR "Village Health Worker" OR "Village Health Workers" OR “Frontline health workers” OR “midwives” OR “health workers”) |
| **EMBASE** | N = 191 |
| MNCH | “Maternal Health"/exp OR “Maternal Care”/exp OR "newborn"/exp OR “newborns”/exp OR “reproductive health”/exp OR "Family planning services"/exp OR “adolescence”/exp OR “pregnancy”/exp OR “birth”/exp OR “child birth”/exp OR “obstetric care”/exp OR “postpartum”/exp OR “postnatal”/exp OR "Neonate"/exp OR "Neonates"/exp OR "infant"/exp OR "infants"/exp OR “Child Health Services”/exp OR “Child Health”/exp OR “IMCI”/exp OR “eIMCI”/exp OR “e-IMCI”/exp |
| mHealth | “mobile technology”/exp OR “mobile technologies”/exp OR “mhealth”/exp OR “m-health”/exp OR “sms”/exp OR “short message service”/exp OR “text messages”/exp OR “texting”/exp OR “mobile phone”/exp OR “mobile phone technologies”/exp OR “mobile phone technology”/exp OR “wireless”/exp OR “wireless technologies”/exp OR “wireless technology”/exp OR “cellular phone”/exp OR “call”/exp OR “calling”/exp OR “PDA”/exp |
| CHW | “community care”/exp OR “community health worker”/exp OR “chw”/exp OR “chws”/exp OR “village health worker”/exp OR “village health workers”/exp OR “Front health workers”/exp OR “midwives”/exp |
